# CpG and Non-CpG Methylation in Epigenetic Gene Regulation and Brain Function

**DOI:** 10.3390/genes8060148

**Published:** 2017-05-23

**Authors:** Hyun Sik Jang, Woo Jung Shin, Jeong Eon Lee, Jeong Tae Do

**Affiliations:** Department of Stem Cell and Regenerative Biotechnology, KU Institute of Science and Technology, Konkuk University, Seoul 143-701, Korea; huyndig@naver.com (H.S.J.); bobo5369@naver.com (W.J.S.); onyx62@naver.com (J.E.L.)

**Keywords:** epigenetics, CpG methylation, non-CpG methylation

## Abstract

DNA methylation is a major epigenetic mark with important roles in genetic regulation. Methylated cytosines are found primarily at CpG dinucleotides, but are also found at non-CpG sites (CpA, CpT, and CpC). The general functions of CpG and non-CpG methylation include gene silencing or activation depending on the methylated regions. CpG and non-CpG methylation are found throughout the whole genome, including repetitive sequences, enhancers, promoters, and gene bodies. Interestingly, however, non-CpG methylation is restricted to specific cell types, such as pluripotent stem cells, oocytes, neurons, and glial cells. Thus, accumulation of methylation at non-CpG sites and CpG sites in neurons seems to be involved in development and disease etiology. Here, we provide an overview of CpG and non-CpG methylation and their roles in neurological diseases.

## 1. Introduction

Epigenetic marks include a variety of gene regulatory events, such as chromatin structure remodeling, histone modifications, DNA methylation, and small noncoding RNAs, that do not entail changes in the DNA sequence [1]. Gene expression is directly associated with RNA polymerase and transcription factors that bind to regulatory sequence elements, such as promoters and enhancers. Epigenetic events regulate gene expression at both transcription (histone modification and DNA methylation) and translation (small noncoding RNA) levels. Specifically, epigenetic regulation is involved in genomic imprinting, X chromosome inactivation, and gene silencing. It is thus closely correlated with disease mechanisms [2].

**Figure 1:**
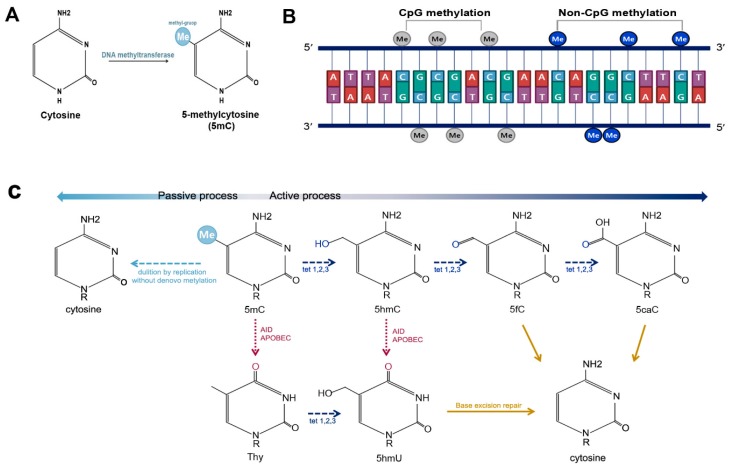
DNA methylation and demethylation. (**A**) DNA methylation occurs at the fifth carbon of cytosine and leads to the formation of 5-methylcytosine (5mC); (**B**) DNA methylation is predominantly found at CpG sites, and is much less commonly observed at non-CpG sites, such as CpA, CpT, and CpC; and (**C**) 5mC can be demethylated by passive or active processes. Active DNA demethylation can occur either via oxidation or deamination. The oxidation process is carried out by Ten-Eleven-Translocation (TET) proteins, including TET1, TET2, and TET3. TETs convert 5mC into 5-hydroxymethylcytosine (5hmC), which is further changed into 5-formylcytosine (5fC) and 5-carboxylcytosine (5caC). 5caC is excised and replaced via base excision repair. 5mC and 5hmC can also be demethylated via deamination by activation induced cytidine deaminase (AID)/apolipoprotein B mRNA editing enzyme, catalytic polypeptide (APOBEC).

In 1975, two independent research groups suggested that the methylation of cytosine might play a pivotal role as an epigenetic mark in animals [3,4]. DNA methylation was found to occur predominantly on cytosines followed by guanine residues (CpG). This type of methylation is referred to as CpG methylation, and cytosine methylated at the fifth carbon of the pyrimidine ring is called 5-methylcytosine (5mC) (Figure 1A,B). The major function of DNA methylation is the suppression of gene expression. Subsequently, DNA methyltransferases (DNMTs), which are specific enzymes that cause methylation at CpG sites, were identified. These enzymes include DNMT1, DNMT3A, and DNMT3B [5].

DNA methylation is also found at sites other than CpG sequences. This type of methylation is referred to as non-CpG methylation, and includes methylation at cytosines followed by adenine, thymine, or another cytosine (Figure 1B). Non-CpG methylation was initially described in the plant genome [6]. It is suggested to be prevalent in human embryonic stem cells (ES cells) and brain tissue, and comprises 0.02% of total methyl-cytosine in differentiated somatic cells [7,8]. Non-CpG methylation is catalyzed by DNMT3A and DNMT3B [7,9,10,11]. However, the exact mechanisms of non-CpG methylation are as yet poorly understood.

On the other hand, 5mC can also be demethylated via either passive or active DNA demethylation. Passive demethylation is carried out by DNA synthesis without de novo methylation, by which 5mC is diluted following replication. Active demethylation entails the modification of the cytosine base via deamination and/or oxidation [12] (Figure 1C). Deamination of cytosine by activation induced cytidine deaminase (AID)/apolipoprotein B mRNA editing enzyme, catalytic polypeptide (APOBEC) yields a uracil, which results in a thymine-guanine mismatch, followed by DNA repair-mediated replacement of thymine with unmethylated cytosine. Active demethylation through oxidation process is carried out by Ten-Eleven-Translocation (TET) proteins, including TET1, TET2, and TET3. TETs oxidize the 5-methyl group of cytosine and convert 5mC into 5-hydroxymethylcytosine (5hmC). 5hmC is further oxidized into 5-formylcytosine (5fC) and 5-carboxylcytosine (5caC), which can be excised via glycosylation by thymine DNA glycosylase, followed by base excision repair. This results in unmethylated cytosine [13,14]. Recent studies have revealed that 5hmC is not only an intermediate in the process of active DNA demethylation, but is also a key element in the regulation of gene expression and chromatin structure [15].

In this review, we will provide an overview of CpG and non-CpG methylation and their functions in brain cells, and neurodevelopmental and neurodegenerative diseases.

## 2. CpG and Non-CpG Methylation in Epigenetic Gene Regulation

### 2.1. DNA Methylation

Epigenetic marks have important roles as regulators of gene expression, which is basically regulated by histone modifications and DNA methylation [16]. DNA methylation is a well-studied epigenetic mechanism that regulates gene expression by adding or removing a methyl group at the fifth carbon of the pyrimidine ring of cytosine to form 5mC [17]. DNA methylation has significant effects on cell development and differentiation [18,19]. In fact, 5mC is a highly conserved epigenetic mark among plants and animal [17]. Most 5mCs are found at CpG sites, where 70–80% of cytosines are methylated. For example, the average methylation rate of *Dnmt1* alleles is 62.2% in wild-type mouse ES cells, 66% in mouse liver, and 81.6% in mouse lung [11]. In human brain, 6–8% of CpG islands in genomic DNA are methylated [20].

Most 5mCs are found in repetitive sequences, gene bodies, and intergenic regions [16,17,21,22]. Repetitive sequences comprise more than 40% of the genome and gene body methylation plays a role in regulating gene expression. Some intergenic regions located between genes also control the expression of genes nearby [23]. For instance, if CpG sites located in a gene body are hypermethylated, gene expression is increased [22]. On the other hand, if CpG sites located in promoters or enhancers are hypermethylated, these regions become heterochromatic and are not bound by transcription activators. This leads to transcriptional silencing [8,24,25]. Therefore, CpG methylation is crucial for gene repression and expression. The correlation between DNA methylation at CpG sites and inhibition of gene expression was first shown in a study of CpG islands, which are regions with relatively high frequencies of CpG dinucleotides. CpG islands in promoter regions of active genes were shown to be unmethylated when compared to coding regions [5]. Recent studies have identified regions located near (within 2 kb) of traditional CpG islands, named CpG shores [26,27,28,29]. CpG shores are related to tissue- and cancer-related methylation and age-related hypomethylation changes [30].

DNMTs are enzymes involved in the transfer of methyl groups to cytosines in DNA. Briefly, methyl groups from the cofactor S-adenosylmethionine are transferred to the 5th carbons of cytosines by DNMTs [5]. Dnmt1, Dnmt3a, and Dnmt3b, which are DNMT family members, have N-terminal regulatory domains and C-terminal catalytic domains [31]. Dnmt1 is essential for the maintenance of methylation and chromatin stability [32,33,34], and Dnmt3a and Dnmt3b act as de novo methyltransferases, and are important for DNA methylation in the early embryonic stages [4,35,36]. DNMT1 is especially highly expressed in postmitotic neurons in the central nervous system, and is involved in neuronal differentiation, migration, and central neuronal connections [37]. Mutations in DNMT1 are associated with hereditary sensory neuropathy dementia [37]. DNMT3L does not contain a conserved motif, but is indispensable for genomic imprinting in oocytes (DNMT3L works in association with DNMT3) [38,39]. DNMT1o is a specific enzyme that is expressed in mouse oocytes and is involved in the maintenance of DNA methylation and imprinting [32,33,34,38,39].

### 2.2. Non-CpG Methylation

In mammals, DNA methylation at CpG dinucleotides is critical for cellular development and differentiation [40,41]. Five-methylcytosines are found primarily at CpG sites, and are found at non-CpG sites, such as CpA, CpT, and CpC. We now know that DNA methylation occurs universally at non-CpG sites, although the function and mechanisms of this type of methylation are not yet elucidated and are still controversial [19,42]. Some researchers believe that non-CpG methylation is a by-product of the hyperactivity of non-specific de novo methylation of CpG sites [19,43]. Others argue that non-CpG methylation is correlated with gene expression and tissue specificity [44,45,46]. For instance, in patients with type 2 diabetes mellitus, the promoter of the *peroxisome proliferator-activated receptor γ coactivator 1α* (*PGC-1 α*) gene is more methylated at non-CpG sites than in healthy controls. This results in the downregulation of *PGC-1 α* [44]. A correlation between non-CpG methylation and transcriptional repression has also been suggested in brain cells [47]. Other reports indicate that non-CpG methylation levels are generally low in promoter regions, although high levels of non-CpG methylation in promoter regions are correlated with gene repression [48,49,50].

**Figure 2:**
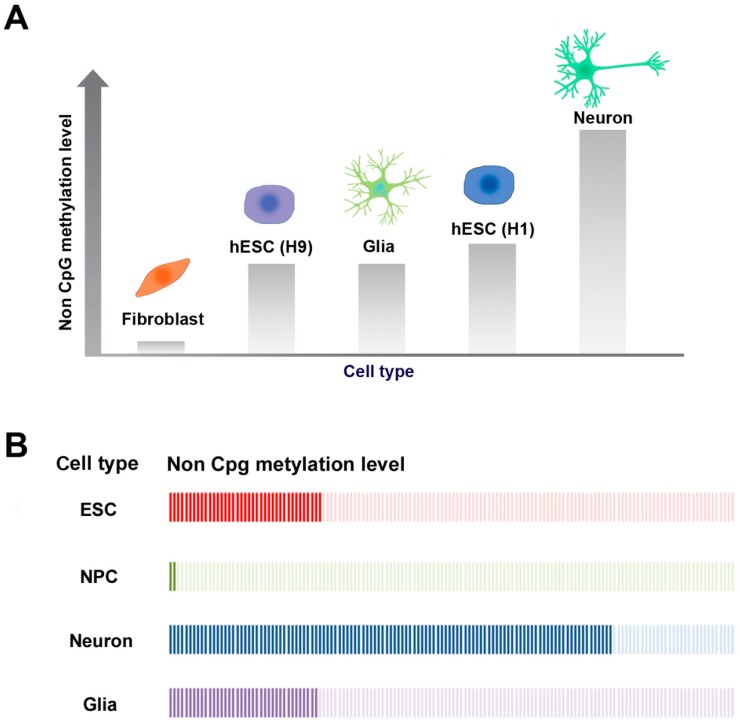
Non-CpG methylation levels in different cell types during differentiation of human ES cells. (**A**) Tissue-specific non-CpG methylation levels. In mammalian somatic cells, neurons have the highest non-CpG methylation levels, although non-CpG methylation is rare in most other differentiated cell types, such as fibroblasts. Non-CpG methylation is also enriched in ES cells. The human male ES cell line (H1) is more highly methylated than the female ES cell line (H9); (**B**) Dynamics of non-CpG methylation levels during the differentiation of ES cells. Non-CpG methylation levels decrease when ES cells differentiate into neural progenitor cells (NPCs). When NPCs are further differentiated into neurons and glial cells, non-CpG methylation is increased again. Interestingly, non-CpG methylation levels are higher in neurons than in glial cells.

Recent studies have revealed that non-CpG methylation is enriched in ES cells [7,8], induced pluripotent stem cells (iPS cells) [45,46], somatic cell nuclear transfer-derived ES (SCNT-ES) cells [46], oocytes [51,52], neurons, and glial cells [9], although it is rare in most differentiated cell types (Figure 2A). Interestingly, neurons accumulate non-CpG methylation during development [47,49,53]. Non-CpG methylation is nearly absent from adult somatic cells and accounts for only 0.02% of the overall 5mCs in somatic cells. On the other hand, there are differences in non-CpG methylation in human pluripotent cell types: human male ES cells (H1 cell line) are heavily methylated up to approximately 25% at non-CpG sites [7,8], but human female ES cells (H9 cell line) are less methylated compared to H1 ES cells at both CpG and non-CpG sites [7,8,54] (Figure 2A). This phenomenon may be explained by the fact that the expression levels of DNMTs are reduced in female ES cells, which would then lead to a reduction in de novo methylation [54]. In both mice and humans, adult brain tissue displays genome-wide non-CpG methylation [9,47,49,53]. Interestingly, different types of brain cells have different levels of non-CpG methylation. Neurons have considerably higher levels of non-CpG methylation than glial cells [9].

Among the different types of non-CpG methylation (CpA, CpT, and CpC), methylation is most common at CpA sites. For instance, in human iPS cells, 5mCs are found in approximately 68.31%, 7.81%, 1.99%, and 1.05% of CpG, CpA, CpT, and CpC sites, respectively [43]. In addition, methylation of 5’-CAG-3’ sequences predominantly occurs in ES cells. This is referred to as CAG methylation [7,8,43]. In contrast, methylation at 5’-CAC-3’ sequences usually occurs in neurons. This is referred to as CAC methylation [7,9,49]. CAG methylation is also found in oocytes, polar bodies, and pluripotent stem cells, such as ES cells, SCNT-ES cells, and iPS cells [45,46,51]. CAC methylation is found in the frontal cortex, neurons, glial cells, and diverse human tissues [9,49].

In mammals, DNA methylation in somatic cells is carried out by DNMT1, DNMT3a, DNMT3b, and DNMT3L [4,17,38]. Non-CpG methylation is carried out by the de novo methyltransferases DNMT3a and DNMT3b, while the maintenance methyltransferase DNMT1 is not associated with non-CpG methylation patterns [11,55]. In previous reports, *Dnmt1* knockout mouse ES cells were shown to retain non-CpG methylation patterns. Compared to *Dnmt1* knockout mice, *Dnmt3l* knockout mice have significantly lower levels of CpA methylation in their prospermatogonia [56]. Furthermore, *Dnmt3a* and *Dnmt3b* double knockout ES cells, where *Dnmt3l* is associated with both *Dnmt3a* and *Dnmt3b* expression, have much lower CpA methylation levels [55,57]. Collectively, non-CpG methylation levels are influenced by de novo DNMTs, such as Dnmt3a, Dnmt3b and Dnmt3l. It is certain that de novo DNMTs affect non-CpG methylation, but their functions and mechanisms in establishing non-CpG methylation are as yet completely unknown.

### 2.3. CpG and Non-CpG Methylation in Brain

According to previous studies, DNA methylation has critical roles in epigenetic regulation of brain development and neurogenesis [58]. DNA methylation may be involved in early brain development and region specification by gene expression [58]. Recently, non-CpG methylation was found at high levels in adult mouse frontal cortex and human brain, while it was rarely detectable in other tissues [9,53]. This fact indicates that extensive de novo DNA methylation occurs during neural maturation in vivo. In fact, mature neurons have significant CpG modifications in response to various stimuli. Non-CpG methylation seems to have different functions in mouse and human brain tissue. Specifically, it is likely to be correlated with gene activity in human brain tissue [8], but is negatively correlated with gene activation in the mouse frontal cortex.

Different neuronal subtypes also have differences in non-CpG methylation patterns in the human brain [59]. The mammalian neocortex contains two major neuronal subtypes: excitatory glutamatergic projection neurons (glutamatergic neurons) and gamma-aminobutyric acid (GABA) ergic interneurons. These neuronal types comprise about 80% and 20% of all neurons, respectively. These two different neuronal subtypes have significantly different DNA methylation patterns, as well as unique gene expression patterns [59]. Kozlenkov and colleagues analyzed genome-wide DNA methylation patterns from in autopsy specimens. They considered glutamatergic neurons (SOX6^−^/NeuN^+^), GABAergic interneurons (SOX6^−^/NeuN^+^), and glial cells (SOX6^−^/NeuN^−^) separately. This genome-wide analysis indicated that the average level of DNA methylation at CpG sites was higher (about 3–4%) in GABAergic interneurons than in glutamatergic neurons and glial cells. DNA hydroxymethyl cytosine, which is an intermediate molecule on the 5mC demethylation pathway, is also higher in glutamatergic neurons than in GABAergic interneurons.

In the in vivo DNA methylome of the adult mouse dentate gyrus, neurons have about 75% CpG methylation and 25% non-CpG methylation [60]. The non-CpG methylation patterns in the mouse brain are conserved in the human brain. In fact, about 83% of non-CpG methylated genes in humans are orthologous genes that are also observed in the mouse brain [60]. Non-CpG methylation is established during postnatal development of the hippocampus and its levels increase over time. Similarly, non-CpG methylation is scarcely detected in human fetal frontal cortex, but is dramatically increased in later life. This increase in non-CpG methylation occurs simultaneously with synaptic development and increases in synaptic density [9]. In contrast, CpG methylation occurs during early development and does not increase over time [47]. In an in vitro differentiation experiment, researchers found that a specific pattern of non-CpG methylation rarely exists in neural progenitor cells (NPCs), but is abundant in neurons. This indicates that new patterns of non-CpG methylation may be established when NPCs differentiate into neurons and glia [9,61]. This phenomenon was also examined during the differentiation of human ES cells to neural cell types [62,63,64] (Figure 2B). ES cells have abundant non-CpG methylation sites. However, during in vitro differentiation into NPCs, genome-wide demethylation is observed at non-CpG sites [62,63,64,65]. When NPCs are further differentiated into neurons and glial cells, non-CpG methylation is reestablished. Interestingly, the primary methylated sites are different in differentiated vs. non-differentiated neurons. CAGs are the primary non-CpG methylation sites in ES cells, while CACs are the most prevalent non-CpG methylation sites in differentiated neural cells. In addition, non-CpG methylation is more abundant in neurons than in non-neural glia [65] (Figure 2B). These data indicate that non-CpG methylation is likely to be related to functional genes characteristic of cells of neural lineage [64].

Transcriptional repression of non-CpG methylation may be mediated by the interaction between DNA-binding proteins and DNA regulatory elements [66]. The *methyl-CpG binding protein 2* (*MeCP2*) gene is especially highly expressed in the brain and some other tissues, such as lung and spleen. Similar to 5mCs in CpGs, non-CpG methylation can also be recognized by MeCP2 [47]. In brain, *MeCP2* expression is higher in neurons than in glia [67,68], and its protein levels are about 5- to 10-fold higher than in other cell types [69,70]. As MeCP2 is important for brain development and the functions of neurons and glial cells [68], it may act as a bridge between transcription machinery and CpG and/or non-CpG methylation. MeCP2 protein has two domains: the methyl-CpG-binding domain (MBD), which binds to methylated DNA, and a transcriptional repression domain (TBD), which interacts with other transcription factors [71,72]. MeCP2 represses the expression of target genes in collaboration with other cofactor, such as the SIN3 transcription regulator (SIN3A) and histone deacetylases (HDACs), which induce chromatin remodeling and subsequent transcriptional silencing [71,72]. Mutations in *MeCP2* lead to neurological diseases, such as Rett syndrome (RTT), in humans.

### 2.4. 5hmC in Brain and Neural Development

The formation of 5hmC, a 5mC demethylation intermediate, is catalyzed by Fe^2+^- and 2-oxoglutarate-dependent dioxygenase TETs [13,14]. In mammals, 5hmC is present in all tissues, and has various levels in different tissues. The highest levels are found in the brain (ranging from 0.4% to 0.7% of total cytosine content) [73,74,75,76]. The levels of 5hmC in other tissues (such as kidney, lung, or liver) are lower than 0.2%. Accordingly, all three TETs (TET1, TET2, and TET3) are expressed in brain. Although little is known about the role of 5hmC in specific regions of brain and neural differentiation, observation of phenotypes after knockout or knockdown of TET1, TET2, and TET3 indicate that these enzymes are likely to be associated with neuronal differentiation and neural progenitor cell formation [77,78,79,80]. 5hmC quantification analysis indicates that 5hmC levels in cerebellum, cortex, and hippocampus are much higher than in mouse ES cells [81]. Mature neurons have higher 5hmC content than neural progenitors and young neurons, suggesting that 5hmC increases during the maturation of neurons [82]. Human ES cell-derived neural stem cells also have high 5hmC content, which is maintained during further neuronal differentiation in vitro. However, differentiation towards the oligodendrocyte lineage results in the progressive loss of 5hmC staining, indicating that high 5hmC content may be related to neuronal function [83]. In addition, there is a correlation between 5hmC and neuronal differentiation during neural lineage differentiation of ES cells, as indicated by comparative hydroxymethylated DNA immunoprecipitation [84]. Although reduced 5hmC levels were observed in most exons and promoters, some regions associated with neural system functions had increased 5hmC levels.

Genome-wide studies suggest that 5hmC is enriched in gene bodies, promoters, and distal regulatory regions. Importantly, 5hmC levels are increased and enriched preferentially in gene bodies of neuron-related genes during neuronal differentiation [85]. The level of 5hmC in gene bodies is positively correlated with transcription in the brain and in other tissues [86], but is negatively correlated with activity of enhancers and promoter [87].

Interestingly, MeCP2, a 5mC-binding protein, is also bind to the 5hmC. However, high-affinity binding of MeCP2 is specific to the CpA hydroxymethylation (5hmCpA), but not to the 5hmCpG, 5hmCpC, and 5hmCpT [88]. MeCP2 binding may inhibit conversion of 5mC to 5hmC [82]. Other 5hmC-binding proteins, such as Uhrf2, Wdr76, Hyy28 (Thyn1), and Neil1 have been identified in ES cells and neural progenitor cells [89], suggesting a role for 5hmC as an epigenetic regulator. Ascorbic acid is shown to interact with TETs and induce 5hmC generation [90]. High concentrations of ascorbic acid in neurons may explain the functional role of the ascorbic acid-TET interaction in neurons [91].

Although 5hmC is likely to be a stable epigenetic marker and is abundant in the adult brain, the exact mechanistic involvement of 5hmC with neurodevelopmental and neurodegenerative diseases remains unclear. However, many researchers have suggested a potential role of 5hmC in neurological diseases, such as Rett syndrome, autism, Huntington’s disease (HD), and Alzheimer’s disease (AD) [92]. Two subsequent derivatives of 5hmC—5fC and 5caC—also transiently accumulate during neuronal and glial differentiation, but their roles in neural differentiation and neurological disease have not been studied much thus far [93].

## 3. Neurological Disorders Associated with DNA Methylation

DNA methylation has been suggested to play an important role in brain function, especially in memory formation [60,94]. Thus, epigenetic alterations in the mammalian brain result in impairment of brain development and neurodevelopmental disorders [95,96,97]. Recent studies have raised concern regarding the effects of aberrant DNA methylation on the origin and progression of neurodegenerative disorders. As described above, DNA methylation is found in brain, as well as in other tissues. However, specifically high levels of non-CpG methylation have been described in brain.

DNA methylation plays an important role in gene regulation. It is thus not strange that some diseases are caused by abnormal DNA methylation [98]. Mutations in genes coding proteins that recognize DNA methylation or those that are required for normal neural development cause epigenetic disorders. These disorders include RTT, fragile X syndrome (FXS), Rubinstein-Taybi syndrome, Coffin-Lowry syndrome, and alpha-thalassemia mental retardation syndrome. In some cases, changes in histone modification or DNA methylation (hypermethylation or hypomethylation) can cause neurodegenerative disorders, such as Parkinson’s disease (PD), AD, and Huntington’s disease [99,100]. Until recently, mechanistic studies on epigenetic disorders have been focused on CpG methylation, although non-CpG methylation has also been recently studied in neurological diseases [53,65].

### 3.1. Alzheimer’s Disease

Gene-specific DNA methylation alterations are thought to be related to AD and AD animal models [97]. AD is characterized by progressive deficits in cognition and memory caused by neuronal degeneration and neuron loss. It is largely caused by the intracellular accumulation of hyperphosphorylated tau, the extracellular accumulation of β-amyloid protein, and the intraneuronal formation of Lewy bodies (α-synuclein-containing inclusions) [101]. Nicolia et al. examined the DNA methylation status of *interleukin-1β (IL-1β)* and *IL-6* in AD, and found that methylation was increased during the first stages of AD and was decreased thereafter. They also found that the expression of complement C3a receptor 1 (C3ar1) is increased during late-onset AD progression. Only *IL-1β* had a pattern of DNA methylation that correlated with gene expression levels. It was little correlation between DNA methylation and gene expression levels for *IL-6* and *C3ar1* [97]. Thus, it may be possible that other epigenetic modifications are more important in controlling AD-related genes.

Sanchez-Mut et al. analyzed 12 distinct brain regions to identify aberrant DNA methylation changes in AD mouse models. These patterns were also shown to be present in patients with AD [102]. They identified three genes that were hypermethylated in both patients with AD and AD mouse models: *thromboxane A2 receptor* (*TBXA2R*), *sorbin and SH3 domain containing 3* (*SORBS3*), and *spectrin β4* (*SPTBN4*). These data suggest that AD may be caused by aberrant DNA methylation in genes associated with cyclic adenosine monophosphate (cAMP) response element-binding protein (CREB) activation (in TBXA2R), the axon initial segment (in SPTBN4), and synapse formation (in SORBS3).

Intriguingly, a recent report has suggested that there is a relationship between altered mitochondrial DNA (mtDNA) methylation and AD [103]. Blanch et al. has shown that CpG and non-CpG sites in the D-loop region of mtDNA are relatively hypermethylated in the entorhinal cortex and substantia nigra in patients with AD. This suggests that epigenetic modulation of mtDNA may also result in neurodegenerative disorders. However, another study indicates that there are methodological limitations to studying human mtDNA, as the methylation levels of mtDNA are much lower (less than 2%) than originally believed [104].

The association between 5hmC and AD is still controversial. Some studies suggest that there is a decrease in 5hmC levels in hippocampal regions of patients with AD [105,106], while other studies suggest that there is an increase in 5hmC levels in gyri of patients with AD [107,108]. The levels of 5hmC vary among different neural subtypes in AD brain. Thus, region- and cell type-specific 5hmC data are required to more precisely understand the mechanisms of AD.

### 3.2. Rett Syndrome

Rett syndrome is an X-linked neurodevelopmental disorder that usually occurs in girls and has a prevalence of 1 in 10,000 to 15,000 [109,110]. It is characterized by dementia, autism, seizures, microcephaly, progressive motor skill regression, and repetitive hand motions. RTT is commonly caused by a mutation in *MeCP2* [59], which is a transcription factor that target genes related to brain and neural development, such as *brain-derived neurotrophic factor* (*Bdnf*), [33,111], *FK506 binding protein 5* [112], *distal-less homeobox 5 (Dlx5)* [113], *Dlx6* [114], *ubiquitin-protein ligase E3A* [115], and *serum/glucocorticoid regulated kinase 1* [62,63,112]. As RTT is dominant and *MeCP2* is located on the X chromosome, RTT arises mostly in the females and is rare males [116]. The *MeCP2* gene is mutated before birth, but RTT features appear 6–18 months after birth. Although the reason for the delayed onset of RTT symptoms remains unclear, it may be related to the interaction between MeCP2 and non-CpG methylation [117]. Chen et al. suggested that binding of MeCP2 to methylated non-CpG sites is necessary for the proper expression of *Bdnf*, which is a key gene for brain development [117]. It is interesting to note that MeCP2 levels and non-CpG methylation are dramatically increased as the brain matures, and that the symptoms of RTT arise late and become severe during further growth. Interestingly, however, CpG methylation remains almost constant during brain development [64]. MeCP2 binding to methylated CpG before birth does not seems to contribute to the direct regulation of gene expression [117]. These facts may indicate that the delayed onset of RTT is mainly due to the association between non-CpG methylation and MeCP2. Considering that MeCP2, which is associated with Rett syndrome, binds to 5hmC and inhibits the conversion of 5mC to 5hmC [89], the MeCP2-5hmC interaction may be involved in the pathogenesis of Rett syndrome.

### 3.3. Fragile X Syndrome (FXS)

FXS is a common hereditary neurodevelopmental disorder associated with the X-linked gene *FMR1* (*fragile X mental retardation-1*), which is highly expressed in neurons. FMRP (encoded by *FMR1*) binds to target mRNA and inhibits translation, which leads to regulation of the formation of dendrites and synapses [118,119]. The features of this syndromes include mental disability, distinct facial features, an aberrantly large head, and behavioral problems, such as hand-flapping and hand-biting [120]. Patients with FXS have smaller cerebellar vermes than normal individuals [121].

The major cause of FXS is the abnormal expansion of CGG repeats in the 5’ untranslated region of the *FMR1* gene, which results in histone deacetylation and DNA hypermethylation. These DNA changes then lead to the transcriptional silencing of the *FMR1* gene [118,119]. Normal individuals have 6–40 CGG repeats, while individuals with the full mutation have more than 200–230 CGG repeats in the *FMR1* gene [122]. Interestingly, transcriptional reactivation can be induced by treating FXS cells with 5-aza-2′-deoxycytidine (5-aza-dC), which is a DNMT inhibitor, but not by treatment with trichostatin A, which is a histone deacetylase inhibitor. This indicates that DNA methylation changes are the major mechanisms underlying FXS [123,124,125]. DNA demethylation following 5-aza-dC treatment is not random, but is rather restricted to genes containing the enriched Gene Ontology (GO) term, which is involved in anatomical structure development, cell differentiation, and cellular protein modification processes [126]. These genes may be the main target genes in epigenetic therapies for FXS.

### 3.4. Parkinson’s Disease (PD)

PD is universal common neurodegenerative movement disorder [127,128]. The main pathological cause of PD is the loss of dopaminergic (DA) neurons in the substantia nigra of the midbrain and Lewy body formation in neurons. The basic cause of PD is as yet not clear, but it is known that both genetic and environmental factors contribute to the pathology of this disease. Recently, increased α-synuclein levels in DA neurons have been suggested as a precipitating factor for PD [129]. DNA methylation analysis of *synuclein-α* (*SNCA*, which encodes for α-synuclein) from peripheral blood obtained from patients with PD patients indicates hypomethylation of *SNCA* in PD [130]. In addition, α-synuclein levels may be decreased by L-dopa treatment via increased methylation of *SNCA*.

Global DNA methylation analysis in a number of patients with PD has revealed that multiple genes involved in neurogenesis, including *Wnt*, are more hypermethylated in PD brain than in normal brain [131]. These hypermethylated genes (*Wnt*, *forkhead box C1*, *neurogenin 2*, *sprouty RTK signaling antagonist 1*, and *catenin beta 1*) also have reduced protein expression in midbrain DA neurons. Consistent with this finding, *SNCA* levels have been shown to be correlated with those of Dnmt1 PD brain [132]. These reports indicate that changes in DNA methylation of *Wnt* and other neurogenesis-involved genes may be involved in the progression of pathogenesis in PD. An inflammatory cytokine, tumor necrosis factor alpha (TNF-α), also appears to be linked to the loss of DA neurons in patients with PD [133]. Hypomethylation of the *TNF-α* promoter, which increases *TNF-α* expression, may increase susceptibility to TNF-α-mediated inflammation, which may then be followed by apoptosis in neuronal cells. Suppression of *TNF-α* may be mediated by DNA methylation at critical CpG sites of *TNF-α* promoters, which modulate the AP2 and specificity protein 1 transcription factors [134].

Similar to AD, PD is associated with mtDNA methylation. However, in contrast to AD (increased DNA methylation), the D-loop region (both CpG and non-CpG sites) of mitochondrial DNA in the substantia nigra has a loss of methylation in patients with PD when compared to control individuals [103]. It remains to be determined whether mtDNA methylation alters nuclear epigenetics and expression levels or global nuclear genomic changes affect mtDNA methylation.

### 3.5. Huntington’s Disease

HD is an autosomal dominant neurodegenerative disorder characterized by constant movement and cognitive disability [135]. HD is caused by an expanded CAG triplet repeat in the Huntingtin (*Htt*) gene, which is located on chromosome 4p16.3 [136]. The *Htt* gene commonly carries a CAG repeat length of 17–20. Repeat lengths over 35 are considered pathogenic [137]. DNA methylation in cortical tissues may be associated with the age of disease onset [138]. Several studies have shown that changes in DNA methylation are associated with the expression of *Htt* in different HD models and in human HD brain. [139,140,141,142]. McFarland et al. found that Htt protein directly interacts with MeCP2 in HD mouse and cellular models [143]. Increased interaction between mutant Htt and MeCP2 may alter the expression of *Bdnf*, which is a gene that is downregulated in HD. This indicates that mutant Htt may induce transcriptional dysregulation of genes implicated in HD pathogenesis.

Wang et al. have suggested that the global loss of 5hmC in brain tissue may be a novel epigenetic marker for HD [141]. They found that the levels of 5hmC were reduced in both the striatum and the cortex in a mouse model of HD. An HD-related gene, *Adora2a* (*Adenosine A2A receptor*, *A2AR*), whose expression is reduced in patients with HD, is regulated by 5hmC levels. This indicates that 5hmC may be involved in the progression of HD by controlling gene expression [139].

### 3.6. Amyotrophic Lateral Sclerosis

Amyotrophic lateral sclerosis (ALS) is a neurodegenerative disease that involves motor neuron degeneration in the brain, brainstem, and spinal cord [144,145,146]. It is characterized by muscle abnormalities, including muscle spasticity, cramps, atrophy and fasciculation [147,148]. Most cases of ALS are sporadic and do not have clear causes. Aberrant DNA methylation patterns and epigenetic modifications are involved in neurodegenerative diseases [149]. Recent studies suggest that altered 5mC or 5 hmC can affect neurodegeneration, which may be caused by abnormal function of DNMTs [150,151]. Motor neurons in patients with ALS especially have changes in Dnmt1, Dmnt3a, and 5mC levels. 5mC accumulates in nuclei of motor neurons in the spinal cord and motor cortex when Dnmt1 and Dnmt3a are increased in spinal and cortical motor neurons [150]. Increased Dnmt3a might also play a pro-apoptotic role in a neurodegenerative model associated with caspase-3 and p53 [150]. Patients with ALS may have one of several gene mutations, such as those in superoxide dismutase 1, chromosome 9 open reading frame 72, TAR DNA binding protein, and FUS [152]. The major cause of ALS may be the expansion of a hexanucleotide (GGGGCC) repeat in the intron of the *C9orf72* gene [153,154]. There are 2–19 repeats in healthy individuals, although the number of repeats is abnormally expanded to more than 30 copies in patients with ALS. The GGGGCC repeat sequence is located between two CpG islands, which are normally free of DNA methylation in healthy controls, but display abnormal CpG methylation at the repeats in patients with ALS [155]. However, the correlation between hypermethylation in the GGGGCC repeats and ALS is still controversial [155,156,157,158].

## 4. Conclusions

Research on epigenetic modifications, including DNA methylation, and their effects on neurological disorders is a rapidly growing field (Table 1). In fact, DNA methylation has been proven to be useful in understanding various neurological diseases. Thus, specific patterns of DNA methylation may be early biomarkers of disease, and epigenetic drugs, such as DNA demethylation inducers, may be used to treat neurodevelopmental and neurodegenerative disorders [100]. As a result, many researchers have tried to find the therapeutics targeting DNA methylation and the changes in the expression levels of genes related to neural function.

Understanding neurological diseases using the epigenetic approach is as yet in its beginning stages. It is thus not easy to obtain reproducible and reliable data regarding DNA methylation from patients with neurological diseases. This is due to the inaccessibility of brain tissue from patients and normal controls. Interestingly, comparison studies using brain tissue and blood have shown that methylation patterns in the brain are very similar to those found in blood [159]. Therefore, blood might be an alternative of brain tissue when analyzing alterations in DNA methylation [160]. A more promising way to study brain tissue might be to use brain organoids, or small brain tissue formations obtained from iPS cells from patients [161,162].

## Figures and Tables

**Table 1:** Neurological diseases and implications of DNA methylation.

Disease	Patterns	Main Factors	References
Alzheimer’s disease	CpG methylation	Methylated *interleukin-6*	[97]
	CpG methylation	Hypermethylated *TXBA2R*, *SORBS3*, and *SPTBN4*	[102]
	CpG/non-CpG methylation	Hypermethylated mitochondrial DNA	[103]
Rett syndrome	MeCp2 mutation	Mutations in MeCP2 target genes (*Bdnf*, *Fkbp5*, *Dlx5*, *Dlx6*, *UBE3A*, and *Sgk*)	[111,112,113,114,115]
	Non-CpG methylation	Binding of MeCP2 to methylated non-CpG sites	[117]
	MeCP2-5hmC interaction	Inhibition of conversion from 5mC to 5hmC	[89]
Fragile X syndrome	Histone deacetylation and DNA hypermethylation	Abnormal expansion of CGG repeat in 5’ UTR of *FMR1* gene	[121]
Parkinson’s disease	CpG methylation	Hypomethylation of SNCA	[130]
	CpG methylation	Hypomethylation of TNF-αpromoter	[134]
	CpG/non-CpG methylation	Hypomethylation of mitochondrial DNA	[102]
Huntington’s disease	CpG methylation	Neurotrophic factors (Bdnf and A_2_A)	[139]
	CpG methylation	Interaction of mutant Htt with MeCP2	[143]
	5hmC	Regulation of *Adora2a* expression by 5hmC levels	[139]
Amyotrophic lateral sclerosis	CpG methylation	5mC accumulation in motor neurons	[150]
	*DMNT1* transcription	Increased levels of Dnmt3a lead to apoptosis	[150]
	Genetic mutation	Gene mutations in *SOD1*, *C9orf72*, *TARDBP*, and *FUS* genes	[152]
